# The root of the matter: Linking oral health to chronic diseases prevention

**DOI:** 10.1016/j.ijcchd.2025.100574

**Published:** 2025-02-12

**Authors:** Francesca D'Aiuto, Jeanie Suvan, Nisachon Siripaiboonpong, Michael A. Gatzoulis, Francesco D'Aiuto

**Affiliations:** a Birbeck University of London, School of Natural Sciences, London, UK; b Glasgow University, Glasgow, UK; c Department of Periodontology, Faculty of Dentistry, Chulalongkorn University, Bangkok, Thailand; d UCL Eastman Dental Institute, Periodontology Unit, London, UK; e Adult Congenital Heart Centre and National Centre for Pulmonary Hypertension, Royal Brompton & Harefield Hospitals, Guys & St Thomas's NHS Trust and Imperial College, London, UK

**Keywords:** Oral health, Periodontitis, Systemic diseases, Cardiovascular health, Chronic inflammation

## Abstract

Oral health is increasingly recognized as a vital component of general health, influencing various systemic systems. Periodontal diseases, particularly periodontitis, a chronic inflammatory condition affecting the gums and supporting tissues of teeth, have far-reaching implications beyond the oral cavity. Treating periodontitis not only benefits oral health but also plays a crucial role in reducing the burden of these chronic conditions, improving patient outcomes and lowering healthcare costs. Regular screenings for oral health issues, especially in patients with conditions such as cardiovascular disease or diabetes, should become standard practice in medical settings. Additionally, oral health professionals must be empowered to identify early signs of systemic diseases, creating a bidirectional flow of referrals between dentists and physicians. Ultimately, prioritizing oral health not only enhances individual well-being but also serves the greater public health good.

## 1 Introduction

Oral health is increasingly recognized as a vital component of general health, influencing various systemic systems. Periodontal diseases, particularly periodontitis, a chronic inflammatory condition affecting the gums and supporting tissues of teeth, have far-reaching implications beyond the oral cavity. This narrative review explores the impact of poor oral health on systemic diseases, including cardiovascular diseases (CVD), diabetes, rheumatoid arthritis (RA), and cognitive decline, particularly Alzheimer's disease. These associations emphasise the importance of oral health not only for preventing localized infections but also for promoting systemic health and public health initiatives [1].

### 1.1 Periodontitis: A key contributor to oral and systemic health

Periodontitis is a chronic inflammatory disease that affects the supporting structures of the teeth, including the gums, periodontal ligament, and alveolar bone. It is one of the most prevalent oral health conditions globally, and a leading cause of tooth loss in adults [1]. Periodontitis arises from untreated gingivitis, where reversible gum inflammation progresses to irreversible damage to the periodontium, potentially leading to tooth mobility and loss [2].

The primary etiological factor in periodontitis is the accumulation of dental plaque—a biofilm composed of bacteria that adhere to the tooth surface. If plaque is not adequately removed through oral hygiene practices, it mineralizes into calculus, providing an environment conducive to bacterial growth below the gum line [3]. These bacteria trigger the immune system, resulting in the release of pro-inflammatory cytokines such as interleukin-1 (IL-1), tumor necrosis factor-alpha (TNF-α), and matrix metalloproteinases (MMPs), which degrade the connective tissues and alveolar bone surrounding the teeth [4].

Periodontitis is not merely a localized infection confined to the oral cavity; it has far-reaching systemic implications. The chronic inflammation associated with periodontitis contributes to the pathogenesis of several systemic diseases, including cardiovascular disease, diabetes, rheumatoid arthritis, and neurodegenerative conditions like Alzheimer's disease [5]. For instance, increased systemic inflammatory markers, such as C-reactive protein (CRP) and IL-6, are commonly elevated in both periodontitis and these systemic conditions, suggesting shared pathological mechanisms [6].

Given its dual impact on oral and systemic health, early diagnosis, prevention, and treatment of periodontitis are critical. Regular dental visits, coupled with proper oral hygiene practices, can significantly reduce the risk of periodontitis and its systemic consequences. This underlines the importance of integrated care, where dental and medical professionals collaborate to address both oral health and systemic health outcomes [7] (Figure).

**Figure:**
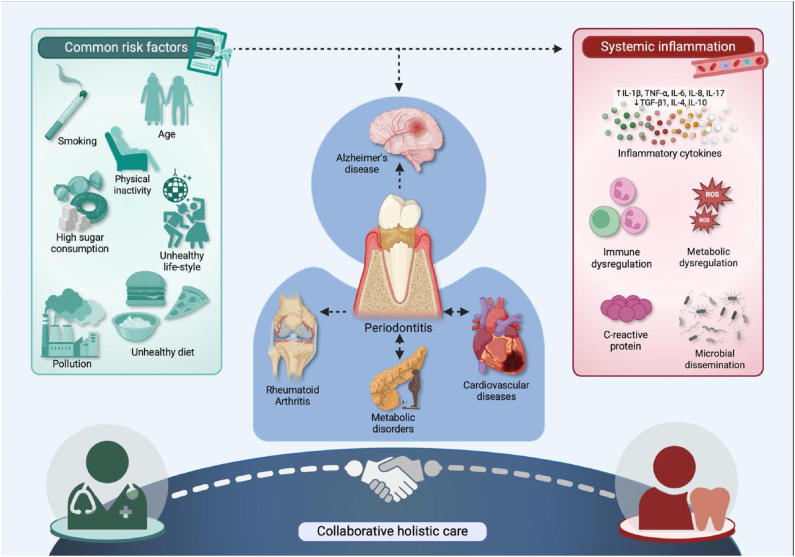

Furthermore, there is epidemiological evidence that suboptimal diet, with excessive intake of extrinsic sugars and fat and low intake of fibre and legume is associated with increased periodontal inflammation [8]. Smoking, poverty, low education level, and limited access to proper dental care have all been postulated as factors aggravating periodontitis [9].

### 1.2 The connection between oral health and cardiovascular diseases

Cardiovascular diseases (CVDs) remain the leading cause of death worldwide, responsible for about 32 % of global deaths annually [10]. A growing body of evidence links poor oral health, particularly periodontitis, to adverse cardiovascular outcomes, including myocardial infarction, stroke, and atherosclerosis [7].

Periodontitis leads to systemic spread of periodontal pathogens, such as *Porphyromonas gingivalis* and *Treponema denticola*, which have been identified in atherosclerotic plaques [11]. These bacteria, entering the bloodstream during routine activities such as brushing or chewing, trigger systemic inflammation that contributes to endothelial dysfunction [6]. This inflammatory state is exacerbated by elevated levels of pro-inflammatory markers, such as C-reactive protein (CRP), tumor necrosis factor-alpha (TNF-α), and interleukin-6 (IL-6), commonly associated with both periodontitis and cardiovascular diseases [4]. Chronic inflammation accelerates atherosclerosis and heightens the risk of cardiovascular events [12].

Importantly, studies have demonstrated that treating periodontitis can lower systemic inflammation, improve endothelial function, and reduce the risk of cardiovascular disease [13]. Therefore, periodontitis is not merely a localized oral condition but a significant contributor to systemic inflammation that affects vasculature and cardiovascular health.

### 1.3 Periodontitis and metabolic disorders: the case of diabetes

Diabetes mellitus, a chronic metabolic disorder, exhibits a strong bidirectional relationship with periodontitis. Individuals with diabetes are more likely to develop periodontitis due to impaired immune responses, poor wound healing, and elevated glucose levels that foster bacterial colonization [3]. Conversely, periodontitis worsens glycemic control in patients with diabetes [14].

Chronic inflammation from periodontitis exacerbates insulin resistance and increases blood glucose levels, further damaging the periodontal tissue [15]. Inflammatory mediators like TNF-α and IL-6 interfere with insulin signalling, complicating glycemic management. Clinical trials show that treatment of periodontitis can lead to a reduction in HbA1c levels by approximately 0.4 % in treated patients [16]. This further highlights the essential role of oral health in diabetes management, reinforcing the need for integrated care between medical and dental professionals.

Effective treatment of periodontitis not only improves glycemic control but also helps prevent the progression of diabetes-related complications such as cardiovascular disease, nephropathy, and retinopathy, further emphasizing the systemic relevance of oral health [5].

### 1.4 Periodontitis and rheumatoid arthritis: A shared inflammatory pathway

The relationship between oral health and systemic inflammatory diseases extends to rheumatoid arthritis (RA), an autoimmune disorder affecting the joints. RA and periodontitis share common inflammatory pathways and risk factors, including smoking and genetic predisposition [17].

Both RA and periodontitis are driven by elevated levels of pro-inflammatory cytokines, such as TNF-α, IL-1, and IL-6, which promote tissue destruction in the joints and periodontium [18]. *Porphyromonas gingivalis*, a key periodontal pathogen, plays a unique role in RA by inducing citrullination, a process that generates antigens involved in RA's autoimmune response [19]. Citrullinated peptides trigger the production of anti-citrullinated protein antibodies (ACPA), a crucial marker in RA pathogenesis.

Periodontal treatment can reduce RA symptoms and systemic inflammation. A study by Nguyen et al. [20] reported a significant reduction in Disease Activity Score-28 (DAS28) in RA patients receiving periodontal treatment. These findings highlight the bidirectional nature of periodontitis and RA, further illustrating the importance of integrated care approaches between rheumatologists and dental professionals.

### 1.5 Cognitive decline and Alzheimer's disease: the oral-brain connection

Emerging evidence suggests a compelling association between poor oral health and cognitive decline, particularly Alzheimer's disease. Chronic periodontitis is linked to an increased risk of cognitive impairment through mechanisms involving systemic inflammation and bacterial dissemination [21].

Pathogens such as *Porphyromonas gingivalis* and its toxic byproducts (gingipains) have been found in the brains of individuals with Alzheimer's disease, contributing to neuroinflammation, a hallmark of Alzheimer's pathology [22]. Chronic systemic inflammation driven by periodontitis also accelerates cognitive decline [23].

A longitudinal study showed that individuals with severe periodontitis had a higher risk of cognitive decline over a 20-year period [24]. This suggests that addressing oral inflammation could play a significant role in preserving cognitive function in older adults.

### 1.6 Public health perspective: economic and social impact of prioritizing oral health

Promoting oral health as a public health priority has significant economic and social implications. Neglecting oral health, particularly the prevention and treatment of periodontitis, increases the burden of systemic diseases, such as cardiovascular disease, diabetes, RA, and cognitive decline.

The global economic burden of oral diseases was estimated at $544 billion annually in 2018 [1]. Poor oral health exacerbates systemic conditions, such as diabetes and cardiovascular diseases, significantly increasing healthcare costs. The prevention of periodontitis through effective oral hygiene and regular dental care can result in substantial healthcare savings by mitigating the complications of these chronic diseases [3].

From a social perspective, addressing oral health disparities can reduce health inequities. Vulnerable populations, including those of lower socioeconomic status and the elderly, often face barriers to accessing dental care, which increases the prevalence of periodontitis and related systemic health burdens [25]. Public health campaigns focused on improving access to preventive dental care and oral hygiene education, in conjunction with appropriate life-style choices such as optimal diet and smoking cessation can significantly reduce these disparities and improve population-wide health outcomes.

## 2 Conclusions

Periodontitis is a chronic inflammatory disease with profound implications for systemic health. Its links to conditions such as cardiovascular disease, diabetes, rheumatoid arthritis, and cognitive decline highlighting the need for a coordinated, multidisciplinary approach to healthcare. Treating periodontitis not only benefits oral health but also plays a crucial role in reducing the burden of these chronic conditions, improving patient outcomes and lowering healthcare costs.

To achieve this, the integration of oral and medical care is essential. One key step is fostering collaboration between dental and medical professionals, ensuring that patients receive holistic care that addresses both oral health and general health. Regular screenings for oral health issues, especially in patients with conditions such as cardiovascular disease or diabetes, should become standard practice in medical settings. Additionally, oral health professionals must be empowered to identify early signs of systemic diseases, creating a bidirectional flow of referrals between dentists and physicians.

Prevention must be at the forefront of these efforts. This includes public health initiatives that promote good oral hygiene practices, regular dental visits, and community-based interventions. Countries around the world can adopt strategies that suit their unique healthcare landscapes but ultimately share the same goal: preventing periodontitis to improve both oral and systemic health outcomes. For instance, countries with well-established healthcare systems may integrate oral health screenings into routine medical care, while those with more limited resources might focus on public health campaigns and increasing access to preventive dental services in underserved communities.

Globally, oral health promotion can also be integrated into broader health promotion campaigns. Public health systems can implement preventive measures, such as fluoridation of water supplies, public education on oral hygiene, and subsidized dental care services. Such efforts have been shown to reduce the incidence of periodontitis and its associated complications (Petersen, 2008). This approach can be tailored by each country to suit local needs, but the underlying principle remains the same: prevention is better than treatment.

Ultimately, prioritizing oral health not only enhances individual well-being but also serves the greater public health good. Governments, healthcare professionals, and public health authorities must work together to ensure that oral health is viewed as an integral part of overall health. By integrating oral care into general healthcare systems and focusing on prevention, the global burden of chronic diseases linked to poor oral health can be significantly reduced, leading to healthier populations and more sustainable healthcare systems.

## CRediT authorship contribution statement

**Francesca D'Aiuto:** Writing – review & editing, Writing – original draft, Data curation, Conceptualization. **Jeanie Suvan:** Writing – review & editing. **Nisachon Siripaiboonpong:** Writing – review & editing. **Michael A. Gatzoulis:** Writing – review & editing, Conceptualization. **Francesco D'Aiuto:** Writing – review & editing, Writing – original draft, Supervision, Project administration, Methodology, Conceptualization.

## Declaration of competing interest

The authors declare the following financial interests/personal relationships which may be considered as potential competing interests:Francesco D'Aiuto reports a relationship with Floe Limited Company that includes: equity or stocks. MG is the Editor in Chief of the IJCCHD but was not involved with the handling of this paper.

If there are other authors, they declare that they have no known competing financial interests or personal relationships that could have appeared to influence the work reported in this paper.
